# Deletion of *AS87_03730* gene changed the bacterial virulence and gene expression of *Riemerella anatipestifer*

**DOI:** 10.1038/srep22438

**Published:** 2016-03-01

**Authors:** Xiaolan Wang, Jiaping Yue, Chan Ding, Shaohui Wang, Beibei Liu, Mingxing Tian, Shengqing Yu

**Affiliations:** 1 Shanghai Veterinary Research Institute, Chinese Academy of Agricultural Sciences (CAAS), Shanghai, P. R. China

## Abstract

*Riemerella anatipestifer* is an important pathogen of waterfowl, which causes septicemia anserum exsudativa in ducks. In this study, an *AS87_03730* gene deletion *R. anatipestifer* mutant Yb2ΔAS87_03730 was constructed to investigate the role of *AS87_03730* on *R. anatipestifer* virulence and gene regulation. By deleting a 708-bp fragment from *AS87_03730*, the mutant Yb2ΔAS87_03730 showed a significant decreased growth rate in TSB and invasion capacity in Vero cells, compared to wild-type strain Yb2. Moreover, the median lethal dose (LD_50_) of Yb2ΔAS87_03730 was 1.24 × 10^7^ colony forming units (CFU), which is about 80-fold attenuated than that of Yb2 (LD_50_ = 1.53 × 10^5^ CFU). Furthermore, RNA-Seq analysis and Real-time PCR indicated 19 up-regulated and two down-regulated genes in Yb2ΔAS87_03730. Functional analysis revealed that 12 up-regulated genes were related to “Translation, ribosomal structure and biogenesis”, two were classified into “Cell envelope biogenesis, outer membrane”, one was involved in “Amino acid transport and metabolism”, and the other four had unknown functions. Polymerase chain reaction and sequence analysis indicated that the *AS87_03730* gene is highly conserved among *R. anatipestifer* strains, as the percent sequence identity was over 93.5%. This study presents evidence that *AS87_03730* gene is involved in bacterial virulence and gene regulation of *R. anatipestifer*.

*Riemerella anatipestifer* is a significant pathogen of waterfowl, turkey and other birds^1^,^2^. It causes epizootic infectious polyserositis in ducks characterized by lethargy, diarrhea, respiratory and nervous symptoms, which led to high mortality and consequently to great economic losses^3^. In view of its veterinary importance, many investigations have been carried out on the virulence factors of *R. anatipestifer*, including CAMP cohemolysin, OmpA, nucleoside-diphosphate-sugar epimerase, and glycosyl transferase etc.^4^,^5^,^6^,^7^. Recently, 49 virulence associated genes were identified by random transponson mutagenesis^8^.

The *luxE* gene from a number of bioluminescent bacteria had been well defined by genetic and biochemical analysis, including *Vibrio harveyi*, *Vibrio fischeri*, *Photobacterium phosphoreum*, etc^9^,^10^,^11^. The *luxE* gene encodes acyl-protein synthetase (LuxE) which activates the fatty acid, results in the formation of a fatty acyl-AMP intermediate and functions as the second step in the bioluminescent fatty acid reduction system^12^. Up to date, most studies focus on the role of *luxE* in bacterial bioluminescence reaction, and the role of *luxE* in *R. anatipestifer* has remained unknown. In this study, a *luxE* homology gene deletion mutant strain Yb2ΔAS87_03730 was constructed by allelic exchange, and the roles of *AS87_03730* gene on bacterial growth, adherence and invasion capability, as well as colonization and development during infection were investigated. Furthermore, the function of *AS87_03730* on the gene regulation at genome level of *R. anatipestifer* was investigated using RNA-Seq, the differentially expressed genes between mutant strain Yb2ΔAS87_03730 and wild-type strain Yb2 were analyzed. The distribution of *AS87_03730* gene in 36 *R. anatipestifer* strains with different serotypes and the gene homology were also analyzed.

## Results

### Characterization of mutant strain Yb2ΔAS87_03730

**Figure 1:**
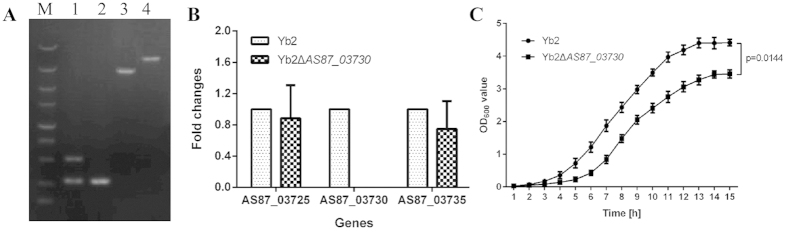
Characterization of mutant strain Yb2ΔAS87_03730. (**A**) PCR amplification. Lane M: DM5000 DNA Marker (CWBIO, Beijing, China). Lane 1: Partial sequences of AS87_03730 gene and 16S rRNA were amplified from *R. anatipestifer* wild-type strain Yb2 using primers AS87_03730 in-F/AS87_03730 in-R and RA 16S rRNA-F/RA 16S rRNA-R, showing a 534-bp fragment of AS87_03730 or a 496-bp fragment of RA 16S rRNA. Lane 2: Partial sequence of *R. anatipestifer* 16S rRNA was amplified from mutant strain Yb2ΔAS87_03730 using primers RA 16S rRNA-F/RA 16S rRNA-R, showing a 496-bp fragment of RA 16S rRNA, no 534-bp fragment of AS87_03730 gene was amplified using primers AS87_03730 in-F/AS87_03730 in-R. Lane 3: A 3,681-bp flanking fragment of *AS87_03730* gene was amplified from wild-type strain Yb2 using primers AS87_03730 out-F/AS87_03730 out-R. Lane 4: A 4,092-bp flanking fragment of *AS87_03730* gene was amplified from mutant strain Yb2ΔAS87_03730 using primers AS87_03730 out-F/AS87_03730 out-R, indicating that a 708-bp fragment of *AS87_03730* gene was replaced with the SpecR cassette. (**B**) Real-time PCR analysis. The transcription levels of upstream *AS87_03725* gene and downstream *AS87_03735* gene were measured. The changes of mRNAs were expressed as fold expression and calculated using the comparative CT (2^−ΔΔCT^) method. No transcription of *AS87_03730* was detected from mutant strain Yb2ΔAS87_03730. Error bars represent SD from three replicates. (**C**) Bacterial growth curves. The mutant strain Yb2ΔAS87_03730 and wild-type strain Yb2 were grown on TSB medium, and growth of each strain was monitored by measuring the OD_600_ values. The bacterial growth rate of mutant strain Yb2ΔAS87_03730 was significantly lower than that of wild-type strain Yb2 (*p* = 0.0144). The data was analyzed using Two-way ANOVA. Error bars represent the standard deviation of three independent experiments.

The *AS87_03730* gene was deleted from the chromosome of *R. anatipestifer* Yb2 by alletic exchange, which was replaced by a Spec^R^ cassette. The mutant strain was screened on TSA plates containing kanamycin (50 μg/ml) and spectinomycin (80 μg/ml), and confirmed by PCR amplification of the *AS87_03730* and 16S rRNA fragments from transconjugants (Fig. 1A). Real-time PCR analysis further confirmed that *AS87_03730* transcription was abolished in the mutant strain; however, inactivation of the *AS87_03730* gene had no effect on the transcription of chromosomally upstream *AS87_03725* and downstream *AS87_03735* genes (Fig. 1B). The *AS87_03730* deletion mutant strain was designated as Yb2ΔAS87_03730.

Mutant strain Yb2ΔAS87_03730 was viable when grown on TSB medium supplemented with kanamycin (50 μg/ml) and spectinomycin (80 μg/ml), however, the growth rate was significantly lower than that of wild-type strain Yb2 (Fig. 1C). When grown on TSA, mutant strain Yb2ΔAS87_03730 formed smooth, slightly raised and non-pigmented colony, which is similar to that of wild-type strain Yb2. Biochemical analysis demonstrated that both mutant strain Yb2ΔAS87_03730 and wild-type strain Yb2 were unable to ferment saccharides (glucose, fructose, galactose, lactose, maltose, sucrose), but produced urease and liquefied gelatin (data not shown). The drug sensitivity of mutant strain Yb2ΔAS87_03730 was also determined, no differences were observed when compared to wild-type strain Yb2 (data not shown). These data suggest that the *AS87_03730* gene has no influence on biochemical property and drug sensitivity of *R. anatipestifer*.

### Deletion of *AS87_03730* gene decreased bacterial adherence and invasion abilities

**Figure 2:**
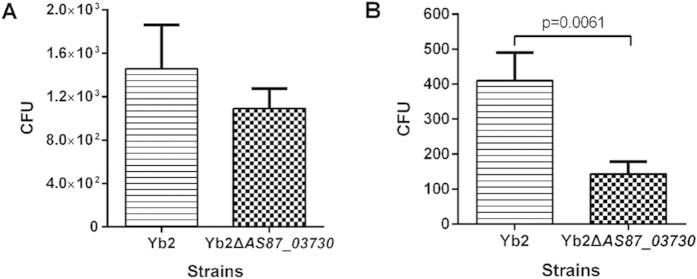
Bacterial adherence and invasion assays. The assays were performed on Vero cells. (**A**) Adherence assay. (**B**) Invasion assay. The Vero cells were infected with mutant strain Yb2ΔAS87_03730 or wild-type strain Yb2 at an MOI of 50, and incubated for 1.5 h to count the number of cell-adherent bacteria. For the invasion assay, the extra-cellular bacteria were killed by incubation of the monolayer with DMEM medium supplemented with 100 μg/ml gentamicin for an additional 1 h. Data were presented as mean ± standard deviations from three independent experiments. The data was analyzed using Student’s t-tests.

The adherence and invasion abilities of mutant strain Yb2ΔAS87_03730 to Vero cells were compared with wild-type strain Yb2 to investigate the role of *AS87_03730* gene on the attachment and invasion of *R. anatipestifer* to host cells. When infection was performed at a multiplicity of infection (MOI) of 50, the adhered bacteria of mutant strain Yb2ΔAS87_03730 was 1,090 CFU/well, which was slightly decreased in comparison with that of wild-type strain Yb2 (1,456 CFU/well). After additional 1 h of incubation with 100 μg/ml gentamicin, the invaded bacterial counts of mutant strain Yb2ΔAS87_03730 was 140 CFU/well, about 3-fold lower than that of wild-type strain Yb2 (410 CFU/well) (*p* < 0.001). The results demonstrated that deletion of *AS87_03730* gene has no influence on the bacterial adherence, but decreased bacterial invades significantly (Fig. 2).

### Determination of the bacterial virulence

Bacterial virulence was evaluated by the median lethal dose (LD_50_) using 18-day-old Cherry Valley ducks. The LD_50_ for mutant strain Yb2ΔAS87_03730 was 1.24 × 10^7^ CFU, which was 80-fold attenuated virulence than that of wild-type strain Yb2 (1.53 × 10^5^ CFU).

**Figure 3:**
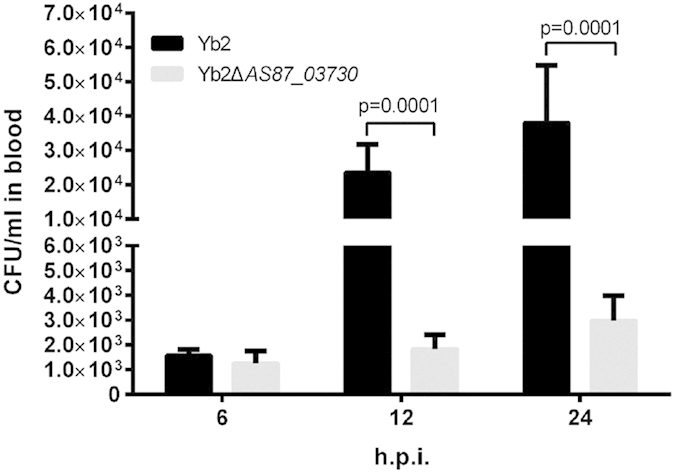
Blood bacterial loadings in *R. anatipestifer* infected ducks. Six ducks were injected intramuscularly with 1 × 10^6^ CFU of each bacterial strain. Blood samples were collected at 6, 12, and 24 h post infection and the bacterial CFU were counted. Data were presented as mean ± standard deviations from six infected ducks. The data was analyzed using Student’s t-tests.

To further investigate the role of *AS87_03730* gene on systemic infection *in vivo*, the bacterial loading in blood of infected ducks was quantified. The bacterial recovery of mutant strain Yb2ΔAS87_03730 was 1,255 CFU/ml, 1,845 CFU/ml, and 2,980 CFU/ml at 6, 12 and 24 h post infection (h.p.i.), respectively, whereas the bacterial recovery of wild-type strain Yb2 was 1,580 CFU/ml at 6 h.p.i., 2.36 × 10^4^ CFU/ml at 12 h.p.i., and 3.80 × 10^4^ CFU/ml at 24 h.p.i. respectively, indicating that mutant strain Yb2ΔAS87_03730 was drastically attenuated (Fig. 3).

### Determination and functional categories of the differentially expressed genes

Differentially expressed genes between mutant strain Yb2ΔAS87_03730 and wild-type strain Yb2 was investigated using strand-specific Illumina RNA-Seq analysis. In total, 31 genes were up-regulated and three genes were down-regulated in mutant strain Yb2ΔAS87_03730, compared to wild-type strain Yb2 (see Supplementary Table S1). Of them, real-time PCR further verified that 19 genes in Yb2ΔAS87_03730 were up-regulated by >2-fold at the transcriptional levels (Table 1). The proteins encoded by these genes were categorized on the basis of their putative functions. The products of up-regulated gene were mostly (63.16%, 12/19) related to “Translation, ribosomal structure and biogenesis”, and two proteins, encoded by *AS87_03970* and *AS87_06085*, were classified into “Cell envelope biogenesis, outer membrane”. The protein encoded by *AS87_02350* was involved in “Amino acid transport and metabolism”. There are only two down-regulated genes for hypothetical proteins, and one is involved in “Defense mechanisms”, the other has unknown function. Pathway analysis for the differentially expressed genes was performed according to the latest KEGG database (http://www.genome.jp/kegg/), and the differentially expressed genes were distributed in clusters. Furthermore, the up-regulated genes mostly (63.16%, 12/19) occurred within ribosome pathway (see Supplementary Figure S1), indicating *AS87_03730* regulates the genes mainly responsible for translation process.

### *AS87_03730* gene is highly conserved in *R. anatipestifer* strains

**Figure 4:**
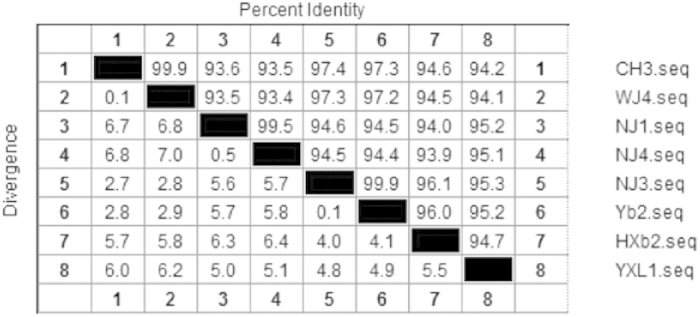
Identity and divergence analysis of *AS87_03730* gene in *R. anatipestifer* strains. The nucleotide sequences of *AS87_03730* from eight *R. anatipestifer* strains with different serotypes were sequenced and analyzed by DNASTAR software for percent identity and divergence. The percent sequence identity was >93.5%.

The *AS87_03730* gene was successfully amplified from all 36 *R. anatipestifer* strains tested. Eight of the PCR products were further subjected to sequence analysis, indicating that Yb2 *AS87_03730* gene shares a sequence identity of more than 93.5% with other 7 strains (Fig. 4). In addition, BLAST analysis showed that Yb2 *AS87_03730* gene exhibits 100%, 100%, 97%, 97% and 91% identity compared to RA-CH-2 (GenBank accession no. CP004020.1), DSM15868 (GenBank accession no. CP002346.1), CH3 (GenBank accession no. CP006649.1), RA-CH-1 (GenBank accession no. CP003787.1) and RA-GD (GenBank accession no. CP002562.1), respectively. These data indicated that *AS87_03730* gene was highly conserved in *R. anatipestifer*. Moreover, *AS87_03730* gene shares 71% similarity to the BD94_0721 gene encoding putative acyl protein synthase/acyl-CoA reductase of *Elizabethkingia anopheles* NUHP1 (GenBank accession no. CP007547.1). The predicted LuxE exhibits sequence identity to acyl transferase from other bacteria, including *Chryseobacterium formosense* (GenBank accession no. KFE99559.1; 72%), *Epilithonimonas sp.* FH1 (GenBank accession no. KFC20122.1; 71%), *Elizabethkingia menigoseptica* (GenBank accession no. WP_026149215.1; 69%) and *Bergeyella zoohelcum* (GenBank accession no. WP_002687639.1; 61%).

## Discussion

LuxE is an acyl-protein synthetase found in bioluminescent bacteria, which catalyzes the formation of an acyl-protein thiolester from a fatty acid and protein. In addition to found in the bioluminescence system, a LuxE domain is also found in the *Vibrio cholera* RfbN protein, which is involved in the biosynthesis of the O-antigen component 3-deoxy-L-*glycero*-tetronic acid^13^. In *R. anatipestifer* strain Yb2, the homology gene of *luxE* is *AS87_03730*, the prevalence of the gene in *R. anatipestifer* that do not produce detectable light has led to the question of what role, if any, there is for the activity encoded by the *AS87_03730* gene in non-luminescent bacteria. To better evaluate the role of *AS87_03730* in *R. anatipestifer*, we constructed a mutant strain Yb2ΔAS87_03730, in which the *AS87_03730* gene was inactivated by deleting a 708-bp fragment from the gene in wild-type strain Yb2. The mutant strain Yb2ΔAS87_03730 showed different growth characteristics in TSB, decreased capacity of invasion, and attenuated virulence in ducks.

*R. anatipestifer* infection is characterized as septicemia, hence, this species of bacteria have ability to colonize and develop in the tissues of their host. We found that the bacterial loadings in the blood of ducks infected with mutant strain Yb2ΔAS87_03730 was significantly decreased than that of Yb2 infected ducks. In addition, infection with mutant strain Yb2ΔAS87_03730 resulted in a marked delay in mortality, and more than 80-fold attenuated virulence. Our results indicated that the *AS87_03730* gene is important for *R. anatipestifer* to establish a systemic infection. A previous study has revealed that the *lux* gene loci (*luxA*, *luxI*, and *luxR*) of *Vibrio fischeri* play an important role in colonization and development of host light organ^14^.

A report has suggested that the products of *luxI* and *luxR* control the expression of several non-*lux* loci, including one required for *V. fischeri* to remain competitive in mixed symbiotic infections^15^. To investigate whether *AS87_03730* gene regulates the expression of other genes in the genome, RNA-Seq analysis was performed to identify differentially expressed genes in mutant strain Yb2ΔAS87_03730. As a result, 31 genes were up-regulated and three genes were down-regulated in mutant strain Yb2ΔAS87_03730. Real-time PCR verification further confirmed 19 genes were up-regulated by over 2-fold. Among them, 12 were classified into “Translation, ribosomal structure and biogenesis”. The ribosome is responsible and essential for translation process. The bacterial ribosome consists of three rRNA molecules and approximately 55 proteins^16^, components that are put together in an intricate and tightly regulated way. Regulation of gene expression on transcriptional level relies on signals transferred to the RNA polymerase (RNAP) that alter the enzyme’s activity or specificity^17^. Normally, rRNA transcription was affected by the stringent response and growth rate regulation. Stringent control leads to a reduction of stable RNA synthesis in response to amino acid starvation, while the growth rate control leads to adjustment of stable RNA synthesis in response to changes in the nutritional quality of growth medium^18^. In this study, the expressions of 12 rRNA proteins were up-regulated in mutant strain Yb2ΔAS87_03070, indicating that it is attempting to adapt to changing condition as a result of inactivation of *AS87_03730* gene.

Two up-regulated genes (*AS87_03970* and *AS87_06085*) were involved in “Cell envelope biogenesis, outer membrane”. The predicted product of *AS87_03970* is a dTDP-4-dehydrorhamnose 3, 5-epimerase, which is involved in the biosynthesis of dTDP-1-rhamnose, an essential component of the bacterial cell wall^19^. More importantly, dTDP-1-rhamnose is the precursor of L-rhamnose, a saccharide required for the virulence of some pathogenic bacteria^20^. The *AS87_06085* gene encodes a dolichyl-phosphate beta-D-mannosyltransferase, which transfers mannose from Dol-P-mannose to Ser or Thr residues on proteins^21^. In *Saccharomyces cerevisiae*, dolichyl-phosphate beta-D-mannosyltransferase is required *in vivo* for glycosyl phosphatidylinositol membrane anchoring, O mannosylation, and N glycosylation^22^. In glycoprotein biosynthesis of *Candida albicans*, dolichol phosphate mannose synthease is activated by cAMP-mediated protein phosphorylation^23^. Similarly, our results showed that *AS87_03730* gene defects in mutant strain Yb2ΔAS87_03730 up-regulated the expression of dolichyl-phosphate beta-D-mannosyltransferase.

One up-regulated gene *AS87_02350* encoding cystathionine beta-synthase (CBS) was related to “Amino acid transport and metabolism”. CBS, primary found in eukaryotes, is the first enzyme in the transsulfuration pathway, catalyzing the conversion of serine and homocysteine to cystathionine and water^24^. The enzyme uses the cofactor pyridoxal-phosphate (PLP) and can be allosterically regulated by effectors such as the ubiquitous cofactor S-adenosyl-L-methionine^25^. In addition, CBS deficiency leads to changes in protein unfolding^26^. Due to *AS87_03730* gene deletion, the biosynthesis of proteins involved in fatty acid metabolism was stalled in mutant strain Yb2ΔAS87_03730. Disturbance of fatty acid metabolism consequently resulted in up-regulated expression of *AS87_02350*.

In conclusion, for the first time, we demonstrated that the *AS87_03730* gene is involved in bacterial virulence and gene regulation in *R. anatipestifer.* Further investigation is necessary to clarify the regulation mechanisms of the *AS87_03730* gene in *R. anatipestifer*.

## Methods

### Bacterial strains, plasmids and culture conditions

The bacterial strains and plasmids used in this study, and their relevant characteristics, are described in Table 2. *R. anatipestifer* strains were grown on tryptic soy agar (TSA, Difco, USA) at 37 °C in 5% CO_2_ or tryptic soy broth (TSB, Difco). *Escherichia coli* strains were grown at 37 °C on Luria-Bertani (LB) plates or in LB broth. For selective growth of bacterial strains, antibiotics were added at following concentrations: ampicillin (100 μg/ml), kanamycin (50 μg/ml), spectinomycin (80 μg/ml) and chloramphenicol (30 μg/ml). *R. anatipestifer* serotype 2 strain Yb2 was selected to construct an *AS87_03730* gene deletion mutant is because serotype 2 is one of the most prevalent serotypes in china^27^ and Yb2 is a kanamycin resistant (Kan^R^) and spectinomycin sensitive (Spec^S^) strain which is good for the further mutant selection.

### Ethics Statement

One-day-old Cherry Valley ducks were purchased from Zhuanghang duck farm (Shanghai, China) and kept under a controlled temperature (28 to 30 °C). The ducks were housed in cages with a 12-h light/dark cycle and free access to food and water during this study. This study was carried out in strict accordance with the recommendations in the Guide for the Care and Use of Laboratory Animals of the Institutional Animal Care and Use Committee (IACUC) guidelines set by Shanghai Veterinary Research Institute, the Chinese Academy of Agricultural Sciences (CAAS). The protocol was approved by the Committee on the Ethics of Animal Experiments of Shanghai Veterinary Research Institute, CAAS (Permit Number: 13–11). All surgeries were performed under sodium pentobarbital anesthesia, and all efforts were made to minimize suffering.

### Construction of mutant strain Yb2ΔAS87_03730

An *AS87_03730* gene deletion mutant was constructed by allelic exchange through the recombinant suicide plasmid pDS132 as described previously^5^. Briefly, a Spec^R^ cassette (1,119 bp) was amplified from plasmid pFW5 using primers Spec-F and Spec-R. A 1,076 bp left flanking region of *AS87_03730* gene was amplified from *R. anatipestifer* Yb2 genomic DNA using primers AS87_03730 Left-F and AS87_03730 Left-R, and cloned into pGEM®-T easy vector (Promega, Madison, WI, USA). A 1,132 bp of right flanking region of *AS87_03730* gene was amplified using primers AS87_03730 Right-F and AS87_03730 Right-R, and cloned into the above-mentioned plasmid; and then a Spec^R^ cassette was inserted at the *Pst*I site. The recombinant DNA was moved to *Sph*I- and *Sal*I-digested suicide plasmid pDS132 to produce pDS132-AS87_03730-LSR (Spec^R^). Subsequently, the recombinant plasmid pDS132-AS87_03730-LSR was transformed into *R. anatipestifer* strain Yb2 by conjugation. The transconjugants were selected by plating on TSA plates containing 50 μg/ml kanamycin and 80 μg/ml spectinomycin. Deletion of *AS87_03730* gene in the mutant was confirmed by PCR analysis and resulting mutant strain was designated as Yb2ΔAS87_03730.

### Phenotype characteristics of Yb2ΔAS87_03730

To determine whether *AS87_03730* gene deletion would influence the bacterial growth, the growth curves of mutant strain Yb2ΔAS87_03730 and wild-type strain Yb2 were measured as previously described^28^. Briefly, equal amount of each bacterial culture in mid-exponential phase was transferred into fresh TSB medium at a ratio of 1:100(v/v) for growing at 37 °C with shaking at 200 rpm. Optical density at 600 nm (OD_600_) was measured at 1 h intervals for 15 h using a spectrophotometer (BIO-RAD, USA). Bacterial growth rate was expressed as OD_600_ values.

The biochemical test for mutant strain Yb2ΔAS87_03730 and wild-type strain Yb2 was carried out using bacterial biochemical tube (Hangwei, Hangzhou, China) following the manufacturer’s instruction. Both strains were also tested against ten commonly used antibiotic disc (ampicillin, amoxicillin, gentamicin, tetracycline, erythromycin, chloramphenicol, ciprofloracin, rifampicin, polymyxin B and trimethoprim-sulfamethoxazde). Inoculums from the tested bacteria were prepared depending on Kirby-Bauer antibiotic testing^29^.

### Adhesion and invasion assays

Adhesion and invasion assays were performed on Vero cells (ATCC CCL-81) in 24-well plates (Corning, NY, USA). The mutant strain Yb2ΔAS87_03730 and wild-type strain Yb2 were grown to mid-logarithmic phase and washed three times with phosphate-buffered saline (PBS) prior to use. Vero cells (approximately 2.5 × 10^5^ cells) were inoculated with treated bacteria at a multiplicity of infection (MOI) of 50, and incubated at 37 °C with 5% CO_2_ for 1.5 h. The infected cells were rinsed three times with PBS to remove unbound bacteria, and then incubated with 0.1% trypsin (100 μl/well) to lyse the eukaryotic cells. The number of cell-adherent bacteria was counted after dilution and plating. For the invasion assay, the infected cells were further incubated for an additional 1 h with DMEM medium supplemented with 100 μg/ml gentamicin to kill extracellular bacteria. After washed three times with PBS, the infected cells were lysed and the amount of intracellular bacteria was determined. All samples were tested in triplicate, and experiments were repeated three times.

### Determination of the bacterial virulence and survival *in vivo*

To determine whether *AS87_03730* gene deletion had an influence on virulence of *R. anatipestifer*, the bacterial median lethal dose (LD_50_) of mutant Yb2ΔAS87_03730 was determined using 18-day-old Cherry Valley ducks as described and compared with that of wild-type strain Yb2^30^. A total of 64 ducks were divided randomly into eight groups (8 ducks per group) and injected intramuscularly with respective bacterial strain at a dose of 10^4^–10^8^ CFU. Ducks in groups 1–4 were injected with 10^4^, 10^5^, 10^6^ or 10^7^ CFU of Yb2 bacteria, and ducks in groups 5–8 were injected with 10^5^, 10^6^, 10^7^ or 10^8^ CFU of Yb2ΔAS87_03730 bacteria, respectively. The infected ducks which appeared clinical signs (clinical signs include lethargy, diarrhea, rough hair coat, frequent seizure activity, paralysis and no eating or drinking, etc.) were sacrificed humanely with an intravenous injection of sodium pentobarbital at a dose of 120 mg/kg and counted as dead. The infected ducks were clinically observed three times at 8-h intervals daily for a period of 7 days post-challenge. The LD_50_ value was calculated by improved Karber’s method^31^.

The bacterial loadings in the blood of infected ducks were counted to evaluate bacterial survival *in vivo*^32^. Twelve 18-day-old Cherry Valley ducks were divided randomly into two groups, and infected intramuscularly with mutant strain Yb2ΔAS87_03730 or wild-type strain Yb2 at a dose of 10^6^ CFU in 0.5 ml PBS. The blood samples were collected at 6 h, 12 h and 24 h post infection, diluted appropriately and plated on TSA for bacterial counting.

### Bacterial RNA isolation

The mutant strain Yb2ΔAS87_03730 and wild-type strain Yb2 were grown to mid-logarithmic phase, harvested by centrifugation at 10,000 × g for 1 min, and then washed once with PBS for RNA extraction. Total RNA was extracted from bacteria (2.5 × 10^9^ CFU) using Trizol reagent (Invitrogen, Carlsbad, CA, USA), according to the manufacturer’s instructions. All RNA samples were treated with TURBO DNA-free^TM^ kit (Ambion, Grand Island, NY, USA) to remove DNA contamination. Quantification and quality assessment of RNA samples were performed with NanoDrop ND-100 instrument (NanoDrop Technologies, Inc., Wilmington, DE, USA). RNA integrity was checked using 1% agarose gel electrophoresis.

### Illumina sequencing for RNA-Seq

Total RNA quantification and quality were assessed by spectrophotometer, and illumina RNA-Seq libraries were generated as following steps: 1) RNA fragmentation; 2) reverse transcription; 3) double-strand cDNA synthesis with dUTP; 4) cDNA fragmentation and end-repair; 5) 3′end “dA” base addition and illumina adapter ligation; 6) cleavage of the uridine containing strand with Uracial DNA Glycosylase; 7) PCR amplification; 8) size selection and purification^33^. The complete libraries were then quantified with Agilent 2100 Bioanalyzer using DNA 1000 Kit. The DNA fragments in these libraries were denatured with 0.1M NaOH to generate single-strand DNA molecules, loaded onto channels of Illumina flow cell at 8 pM concentration, amplified *in situ* using TruSeq SR Cluster Kit v3-cBot-HS (Illumina), and subsequently sequenced for 100 cycles on Illumina HiSeq 2000 according to the manufacturer’s instruction.

### Differential expression analysis

Image analysis and base calling was performed using Solexa pipeline Version 1.8 (Off-Line Base Caller software, Version 1.8)^34^. Trimmed reads were aligned to *R. anatipestifer* Yb2 genome using TopHat2 software (Version 2.0.9)^35^. Transcript levels were calculated as FPKM (fragments per kilobase cDNA per million fragments mapped). Differential expressed genes were analyzed using Cufflinks software (Version 2.1.1) with fold change (cutoff = 2.0)^36^, and considered statistically significant if the fold change was >2.0 and the *p*-value was <0.05.

### Real-time PCR analysis

Transcriptional levels of differently expressed genes obtained in the transcriptome analysis were further confirmed by real-time PCR. Primers were designed from *R. anatipestifer* Yb2 using primer3 online software Version.0.4.0 (http://bioinfo.ut.ee/primer3-0.4.0/) and described in Supplementary Table S2. RNA samples were extracted and purified as mentioned above. cDNA was synthesized using PrimeScript RT Master Mix (Takara) according to the manufacturer’s protocol, and the resulting cDNAs were diluted 3-fold served as templates. Real-time PCR reaction was carried out in Go Taq qPCR^®^ Master Mix (Promega, Fitchburg, WI, USA), and subjected to the Mastercycler ep realplex4 apparatus (Eppendorf, Germany). For each gene, reactions were performed for three RNA samples and each reaction was performed in triplicate. Quantification of transcriptional level was calculated according to the 2^−ΔΔCt^ method using RA 16S rRNA gene for normalization^37^.

### Distribution and sequence analyses of *AS87_03730* gene in *R. anatipestifer* strains

*AS87_03730* gene in *R. anatipestifer* strains was amplified using PCR. Genomic DNAs of 36 *R. anatipestifer* strains with different serotypes were extracted using TIANamp Bacteria DNA kit (Tiangen, Beijing, China). The primers AS87_03730 orf-F and AS87_03730 orf-R were used to amplify the *AS87_03730* sequence using *Premix LA Taq*® (loading dye mix) (Takara, Dalian, China) according to the following cycle parameters: 94 °C for 4 min, 30 cycles of 94 °C for 40 s, 52 °C for 40 s and 72 °C for 1 min, followed by one cycle of 72 °C for 10 min. To investigate *AS87_03730* homology among different *R. anatipestifer* strains, the PCR products from 8 strains were cloned into pGEM®-T easy vector (Promega, Madison, WI, USA), respectively. DNA sequencing was performed on an Applied Biosystems DNA sequencer (ABI 3730XL) by Thermo Fisher Scientific Inc. (Thermo Fisher Scientific, Shanghai, China). The *AS87_03730* gene sequences from these *R. anatipestifer* strains have been submitted to the GenBank database under the accession numbers KM676074 ∼KM076081. Homology of the *AS87_03730* gene sequences was analyzed with DNASTAR software (DNASTAR Inc., Madison, WI, USA).

### Statistical analysis

The statistical significance of the data was determined by the Student’s *t*-test in the SPSS Version 17.0 software (SPSS Inc., Chicago, IL, USA). A *p* value of <0.05 was considered to be statistically significant.

### Nucleotide sequence accession numbers

The accession number for *R. anatipestifer* strain Yb2 in the GenBank is CP007204.

## Additional Information

**How to cite this article**: Wang, X. *et al.* Deletion of *AS87_03730* gene changed the bacterial virulence and gene expression of *Riemerella anatipestifer. Sci. Rep.* **6**, 22438; doi: 10.1038/srep22438 (2016).

## Supplementary Material

Supplementary Information

## Figures and Tables

**Table 1:** Real-time PCR verification of differentially expressed genes in mutant strain Yb2ΔAS87_03730.

Gene locus^a^	Description of genes	Subcellular location^b^	Function group (COGs)^c^	2^—ΔΔCt^^e^
*AS87_05650*	hypothetical protein	Cytoplasmic	–d	4.31
*AS87_04530*	50S ribosomal protein L10	Unknown	COG0244 J	4.30
*AS87_06160*	50S ribosomal protein L28	Cytoplasmic	COG0227 J	4.25
*AS87_03970*	dTDP-4-dehydrorhamnose 3,5-epimerase	Cytoplasmic	COG1898 M	4.19
*AS87_07745*	50S ribosomal protein L23	Cytoplasmic	COG0089 J	3.88
*AS87_07845*	50S ribosomal protein L36	Unknown	–	3.76
*AS87_06455*	50S ribosomal protein L21	Cytoplasmic	COG0261 J	3.54
*AS87_06085*	dolichyl-phosphate beta-D-mannosyltransferase	Cytoplasmic	COG0463 M	3.45
*AS87_07850*	30S ribosomal protein S13	Cytoplasmic	COG0099 J	3.31
*AS87_07760*	50S ribosomal protein L22	Cytoplasmic	COG0091 J	3.22
*AS87_02350*	cystathionine beta-synthase	Cytoplasmic	COG0031 E	2.82
*AS87_07855*	30S ribosomal protein S11	Cytoplasmic	COG0100 J	2.53
*AS87_07860*	30S ribosomal protein S4	Cytoplasmic	COG0522 J	2.24
*AS87_07775*	50S ribosomal protein L29	Unknown	–	2.13
*AS87_00900*	hypothetical protein	Cytoplasmic	–	2.08
*AS87_07790*	50S ribosomal protein L24	Cytoplasmic	COG0198 J	2.04
*AS87_07830*	50S ribosomal protein L15	Cytoplasmic	COG0200 J	2.01
*AS87_07815*	50S ribosomal protein L18	Cytoplasmic	COG0256 J	2.01
*AS87_07805*	30S ribosomal protein S8	Cytoplasmic	COG0096 J	2.00
*AS87_05175*	30S ribosomal protein S12	Cytoplasmic	COG0048 J	1.97
*AS87_04540*	50S ribosomal protein L11	Cytoplasmic	COG0080 J	1.88
*AS87_05170*	30S ribosomal protein S7	Cytoplasmic	COG0049 J	1.78
*AS87_02865*	hypothetical protein	Cytoplasmic	–	1.75
*AS87_04500*	hypothetical protein	Unknown	–	1.72
*AS87_07455*	membrane protein	Unknown	–	1.65
*AS87_07825*	50S ribosomal protein L30	Cytoplasmic	COG1841 J	1.65
*AS87_07870*	50S ribosomal protein L17	Cytoplasmic	COG0203 J	1.57
*AS87_01795*	membrane-binding protein	Unknown	–	1.55
*AS87_05160*	30S ribosomal protein S10	Cytoplasmic	COG0051 J	1.55
*AS87_04930*	glycosyltransferase	Cytoplasmic		1.29
*AS87_02265*	S-adenosylmethionine tRNA ribosyltransferase	Cytoplasmic	COG0809 J	1.09
*AS87_03335*	hypothetical protein	Unknown	–	0.61
*AS87_05990*	hypothetical protein	Unknown	COG1002 V	0.51
*AS87_03730* (mutated gene)	acyl-protein synthetase	Cytoplasmic	–	0.01

**Functional categories: (1) Information storage and processing:** (J: Translation, ribosomal structure and biogenesis; K: Transcription; L: DNA replication, recombination and repair); **(2) Cellular processes and signaling:** (D: Cell division and chromosome partitioning; V: Defense mechanisms; O: Posttranslational modification, protein turnover, chaperones; M: Cell envelope biogenesis, outer membrane; N: Cell motility; T: Signal transduction mechanisms); **(3) Metabolism:** (C: Energy production and conversion; G: Carbohydrate transport and metabolism; H: Coenzyme transport and metabolism; P: Inorganic ion transport and metabolism; E: Amino acid transport and metabolism; F: Nucleotide transport and metabolism; Q: Secondary metabolites biosynthesis, transport and catabolism); **(4) Poorly characterized:** (R: General function prediction only; S: Function unknown).

^a^Based on *R. anatipestifer* Yb2 genome (accession number: CP007204).

^b^Subcellular locations were predicted by the PSORTb Version 3.0 server (http://www.psort.org/).

^c^Functional characterization of the proteins was predicted by searching against eggNOG (Version 3) database using BLASTP.

^d^No related COG.

^e^Results are presented as 2^ΔΔCt^. Figures = 1 indicated that the gene is expressed similarly in both mutant strain Yb2ΔAS87_03730 and wild-type strain Yb2, figures >1 indicated that the gene is over expressed in mutant strain Yb2ΔAS87_03730, and figure <1 indicated that the gene is expressed less in mutant strain Yb2ΔAS87_03730.

**Table 2:** Strains, plasmids and primers used in this study.

Strains, plasmids or primers	Descriptions	Source or reference
Strains		
Yb2	*Riemerella anatipestifer* wild-type strain, serotype 2, virulent strain, Kan^R^	27
Yb2ΔAS87_03730	*AS87_03730* gene deletion Yb2 mutant strain, Spec^R^	This study
CH3, NJ1, NJ4, WJ4, CQ1, CQ3, CQ4, CQ5, NN-2, NN-3, NN-4, YL4, YXb12, YXb14	*Riemerella anatipestifer* wild-type strains, serotype 1	27
NJ3, GD-3, GD-4, GD-5, GD-7, JY1, JY5, SC2, Th4, YXb1	*Riemerella anatipestifer* wild-type strains, serotype 2	27
HXb2, YXL1, YXb11	*Riemerella anatipestifer* wild-type strains, serotype 10	27
NN-6, NN-8	*Riemerella anatipestifer* wild-type strains, serotype 15	This study
NN-7, NN-9, NN-11, GD-1, GD-2, GD-6	*Riemerella anatipestifer* wild-type strains, undefined serotype	This study
Plasmids		
pGEM-T easy vector	TA cloning vector	Promega
pDS132	sacB, mobRP4, oriR6K, Cm^R^	5
pFW5	*E. coli*.-streptococci shuttle vector	5
Primers		
AS87_03730 orf-F	CCGCTCGAGATGCCTTCTATTTTTGATATTA (*Xho*I underlined)	This study
AS87_03730 orf-R	CATGCATGCCTAAGAAACCAAAAGGCTAC (*Sph*I underlined)	This study
AS87_03730 Left-F	CATGCATGCATCCTGAGCTGGGAGAAACA (*Sph*I underlined)	This study
AS87_03730 Left-R	AAACTGCAGCATCTGGGTAGTGCCTGAAC (*Pst*I underlined)	This study
AS87_03730 Right-F	AAACTGCAGGACCATTCCGACATTAGAGG (*Pst*I underlined)	This study
AS87_03730 Right-R	CGCGTCGACGTGATTTCAATAGCCGTTTT (*Sal*I underlined)	This study
Spec-F	AAACTGCAGCGTTCGTGAATACATGTTAT (*Pst*I underlined)	5
Spec-R	AAACTGCAGGCGCTTACCAATTAGAATGA (*Pst*I underlined)	5
AS87_03730 in-F	ACAATTTATAGGCACTCCCG	This study
AS87_03730 in-R	TTAATAGCTCCTGTTCTCCC	This study
AS87_03730 out-F	GCGACCCACTCATAACCC	This study
AS87_03730 out-R	TTCCTAATGGCGACTTTG	This study
RA 16S rRNA-F	CAACCATGCAGCACCTTGAAAA	6
RA 16S rRNA-R	GACGAAAGCGTGGGGAGCGAAC	6

^R^Resistance.

## References

[b1] Glünder G. & Hinz K. H. Isolation of Moraxella anatipestifer from embryonated goose eggs. Avian Pathology 18, 351–355 (1989). 18679866 10.1080/03079458908418608

[b2] Sandhu T. S. & Rimler R. Riemerella anatipestifer infection. Diseases of Poultry 161–166 (1997).

[b3] Sandhu T. in Duck production: science and world practice (ed Farrell D. J. & Stapleton P.) 111–134 (1986).

[b4] Crasta K. C. *et al.* Identification and characterization of CAMP cohemolysin as a potential virulence factor of Riemerella anatipestifer. J. Bacteriol 184, 1932–1939 (2002). 11889100 10.1128/JB.184.7.1932-1939.2002 PMC134935

[b5] Hu Q. *et al.* OmpA is a virulence factor of *Riemerella anatipestifer*. Vet. Microbiol. 150, 278–283 (2011). 21349662 10.1016/j.vetmic.2011.01.022

[b6] Wang X. *et al.* The AS87_04050 gene is involved in bacterial lipopolysaccharide biosynthesis and pathogenicity of *Riemerella anatipestifer*. PloS one 9, e109962 (2014). 25303276 10.1371/journal.pone.0109962 PMC4193840

[b7] Zou J. *et al.* Characterization and cross-protection evaluation of M949_1603 gene deletion *Riemerella anatipestifer* mutant RA-M1. Appl Microbiol Biotechnol. 99, 10107–10116 (2015). 26266750 10.1007/s00253-015-6848-y

[b8] Wang X., Ding C., Wang S., Han X. & Yu S. Whole-genome sequence analysis and genome-wide virulence gene identification of *Riemerella anatipestifer* strain Yb2. Appl Environ Microbiol. 81, 5093–5102 (2015). 26002892 10.1128/AEM.00828-15 PMC4495219

[b9] Johnston T. C., Hruska K. S. & Adams L. F. The nucleotide sequence of the luxE gene of Vibrio harveyi and a comparison of the amino acid sequences of the acyl-protein synthetases from *V. harveyi* and *V. fischeri*. Biochem. Biophys. Res. Commun. 163, 93–101 (1989). 2775296 10.1016/0006-291x(89)92103-7

[b10] Shadel G., Devine J. & Baldvvin T. Control of the lux regulon of *Vibrio fischeri*. J. Biolumin. Chemilumin. 5, 99–106 (1990). 2186599 10.1002/bio.1170050205

[b11] Riendeau D., Rodriguez A. & Meighen E. Resolution of the fatty acid reductase from *Photobacterium phosphoreum* into acyl protein synthetase and acyl-CoA reductase activities. Evidence for an enzyme complex. J. Biol. Chem. 257, 6908–6915 (1982). 7085612

[b12] Lin J. W., Chao Y. F. & Weng S. F. Nucleotide Sequence and Functional Analysis of the *luxE* Gene Encoding Acyl-Protein Synthetase of the *lux* Operon from *Photobacterium leiognathi*. Biochem. Biophys. Res. Commun. 228, 764–773 (1996). 8941351 10.1006/bbrc.1996.1729

[b13] Morona R., Stroeher U. H., Karageorgo L. E., Brown M. H. & Manning P. A. A putative pathway for biosynthesis of the O-antigen component, 3-deoxy-L-*glycero*-tetronic acid, based on the sequence of the *Vibrio cholerae* O1 *rfb* region. Gene 166, 19–31 (1995). 8529890 10.1016/0378-1119(95)00588-9

[b14] Visick K. L., Foster J., Doino J., McFall-Ngai M. & Ruby E. G. *Vibrio fischeri lux* genes play an important role in colonization and development of the host light organ. J. Bacteriol. 182, 4578–4586 (2000). 10913092 10.1128/jb.182.16.4578-4586.2000 PMC94630

[b15] Callahan S. M. & Dunlap P. V. LuxR-and acyl-homoserine-lactone-controlled non-lux genes define a quorum-sensing regulon in *Vibrio fischeri*. J. Bacteriol. 182, 2811–2822 (2000). 10781550 10.1128/jb.182.10.2811-2822.2000 PMC101990

[b16] Schuwirth B. S. *et al.* Structures of the bacterial ribosome at 3.5 Å resolution. Science 310, 827–834 (2005). 16272117 10.1126/science.1117230

[b17] Ishihama A. Functional modulation of *Escherichia coli* RNA polymerase. Annual Reviews in Microbiology 54, 499–518 (2000). 10.1146/annurev.micro.54.1.499 11018136

[b18] Dennis P. P., Ehrenberg M. & Bremer H. Control of rRNA synthesis in *Escherichia coli*: a systems biology approach. Microbiol. Mol. Biol. Rev. 68, 639–668 (2004). 15590778 10.1128/MMBR.68.4.639-668.2004 PMC539008

[b19] Gaugler R. W. & Gabriel O. Biological mechanisms involved in the formation of deoxy sugars VII. Biosynthesis of 6-deoxy -L-talose. J. Biol. Chem. 248, 6041–6049 (1973). 4199258

[b20] Giraud M. F., Leonard G. A., Field R. A., Berlind C. & Naismith J. H. RmlC, the third enzyme of dTDP-L-rhamnose pathway, is a new class of epimerase. Nat. Struct. Biol. 7, 398–402 (2000). 10802738 10.1038/75178

[b21] Tanaka N. *et al.* Characterization of O-mannosyltransferase family in *Schizosaccharomyces pombe*. Biochem. Biophys. Res. Commun. 330, 813–820 (2005). 15809069 10.1016/j.bbrc.2005.03.033

[b22] Orlean P. Dolichol phosphate mannose synthase is required *in vivo* for glycosyl phosphatidylinositol membrane anchoring, O mannosylation, and N glycosylation of protein in *Saccharomyces cerevisiae*. Mol. Cell. Biol. 10, 5796–5805 (1990). 2146492 10.1128/mcb.10.11.5796-5805.1990 PMC361358

[b23] Arroyo-Flores B. L., Calvo-Méndez C., Flores-Carreón A. & López-Romero E. Biosynthesis of glycoproteins in the pathogenic fungus *Candida albicans*: Activation of dolichol phosphate mannose synthase by cAMP-mediated protein phosphorylation. FEMS Immunol. Med. Microbiol. 45, 429–434 (2005). 16055313 10.1016/j.femsim.2005.05.016

[b24] Jhee K. H. & Kruger W. D. The role of cystathionine β-synthase in homocysteine metabolism. Antioxid Redox Sign 7, 813–822 (2005). 10.1089/ars.2005.7.813 15890029

[b25] Banerjee R., Evande R., Kabil Ö., Ojha S. & Taoka S. Reaction mechanism and regulation of cystathionine β-synthase. BBA-Proteins Proteom 1647, 30–35 (2003). 10.1016/s1570-9639(03)00044-x 12686104

[b26] Hnízda A., Jurga V., Raková K. & Kožich V. Cystathionine beta-synthase mutants exhibit changes in protein unfolding: conformational analysis of misfolded variants in crude cell extracts. J. Inherit. Metab. Dis. 35, 469–477 (2012). 22069143 10.1007/s10545-011-9407-4 PMC3319881

[b27] Hu Q. *et al.* Epidemiology investigation and study on the Riemerella anatipestifer infection in Jiangsu and Anhui provinces. Chinese Journal of Veterinary Science and Technology 31, 12–13 (2001).

[b28] Peñuelas-Urquides K., Villarreal-Treviño L., Silva-Ramírez B., Rivadeneyra-Espinoza L. & Said-Fernández S. Measuring of Mycobacterium tuberculosis growth. A correlation of the optical measurements with colony forming units. Braz J Microbiol 44, 287–290 (2013). 24159318 10.1590/S1517-83822013000100042 PMC3804212

[b29] Boyle V. J., Fancher M. E. & Ross R. W. Rapid, modified Kirby-Bauer susceptibility test with single, high-concentration antimicrobial disks. Antimicrob. Agents Chemother. 3, 418–424 (1973). 4790600 10.1128/aac.3.3.418 PMC444425

[b30] Hu Q. *et al.* Characterization of biofilm formation by *Riemerella anatipestifer*. Vet. Microbiol. 144, 429–436 (2010). 20226600 10.1016/j.vetmic.2010.02.023

[b31] Gu B., Li Y., Yu R. & Wang X. Summary of median lethal dose and its calculation methods. China Occupational Medicine 36, 507–508, 511 (2009).

[b32] Wang X., Hu Q., Han X., Ding C. & Yu S. Screening of serotype 2 *Riemerella anatipestifer* candidate strains for the production of inactivated oil-emulsion vaccine. *Chinese* Journal of Animal Infectious Diseases 20, 5 (2012).

[b33] Zhong S. *et al.* High-throughput illumina strand-specific RNA sequencing library preparation. Cold Spring Harb Protoc 2011, pdb. prot5652 (2011). 10.1101/pdb.prot5652 21807852

[b34] Whiteford N. *et al.* Swift: primary data analysis for the Illumina Solexa sequencing platform. Bioinformatics 25, 2194–2199 (2009). 19549630 10.1093/bioinformatics/btp383 PMC2734321

[b35] Kim D. *et al.* TopHat2: accurate alignment of transcriptomes in the presence of insertions, deletions and gene fusions. Genome biol 14, R36 (2013). 23618408 10.1186/gb-2013-14-4-r36 PMC4053844

[b36] Trapnell C. *et al.* Transcript assembly and quantification by RNA-Seq reveals unannotated transcripts and isoform switching during cell differentiation. Nat. Biotechnol. 28, 511–515 (2010). 20436464 10.1038/nbt.1621 PMC3146043

[b37] Huggett J., Dheda K., Bustin S. & Zumla A. Real-time RT-PCR normalisation; strategies and considerations. Genes Immun 6, 279–284 (2005). 15815687 10.1038/sj.gene.6364190

