# Supplementary material for: Deletion of AS87_03730 gene changed the bacterial virulence and gene expression of Riemerella anatipestifer
Source: Sci Rep. 2016 Mar 1;6:22438. doi: 10.1038/srep22438 (PMC4772473; doi:10.1038/srep22438)
Supplement: Supplementary Information [file srep22438-s1.pdf]

Supplementary Information

Deletion of *AS87_03730* gene changed the bacterial virulence and gene expression of *Riemerella anatipestifer*

Xiaolan Wang¶, Jiaping Yue¶, Chan Ding, Shaohui Wang, Beibei Liu, Mingxing Tian, Shengqing Yu*

Shanghai Veterinary Research Institute, Chinese Academy of Agricultural Sciences (CAAS), Shanghai, P. R. China

* Corresponding authors

E-mail: yus@shvri.ac.cn (S.Y.)

¶These authors contributed equally to these work.

Supplementary Figure

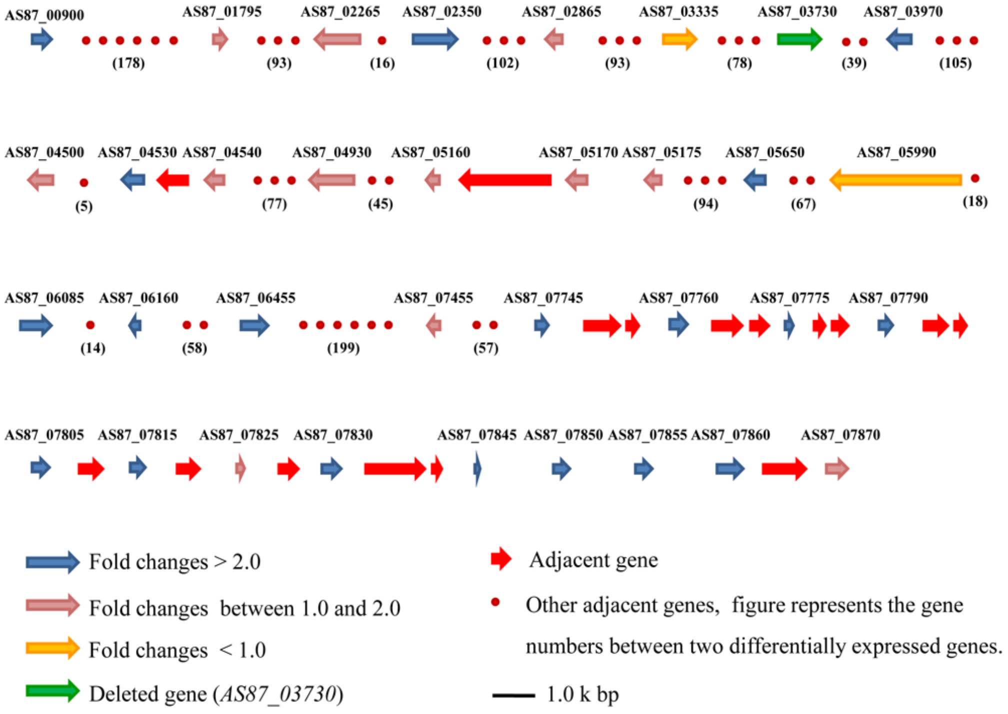

Supplementary Figure S1. Genetic organization of differentially expressed genes. The relative positions of each gene are assigned as predicted by the nucleotide sequence and rely on the same scale. Gene assignments are shown above the rectangles with arrow, and arrow indicates the gene transcriptional direction.

Supplementary Tables

Supplementary Table S1. Differentially expressed genes in mutant strain Yb2Δ*AS87_03730* by RNA-Seq analysis

Gene tag[a]	Fold change	Description of genes
AS87_07455	3.99	membrane protein
AS87_04540	2.92	50S ribosomal protein L11
AS87_07860	3.42	30S ribosomal protein S4
AS87_07745	3.16	50S ribosomal protein L23
AS87_06160	3.29	50S ribosomal protein L28
AS87_03970	3.89	dTDP-4-dehydrorhamnose 3,5-epimerase
AS87_07845	6.80	50S ribosomal protein L36
AS87_04500	3.03	hypothetical protein
AS87_07775	3.98	50S ribosomal protein L29
AS87_01795	3.76	membrane-binding protein
AS87_05175	4.55	30S ribosomal protein S12
AS87_05160	4.24	30S ribosomal protein S10
AS87_07855	4.33	30S ribosomal protein S11
AS87_07830	3.24	50S ribosomal protein L15
AS87_07790	4.78	50S ribosomal protein L24
AS87_07870	3.37	50S ribosomal protein L17
AS87_02865	5.13	hypothetical protein
AS87_06085	3.05	dolichyl-phosphate beta-D-mannosyltransferase
AS87_02265	3.33	S-adenosylmethionine tRNA ribosyltransferase
AS87_06455	2.74	50S ribosomal protein L21
AS87_00900	3.27	hypothetical protein
AS87_07825	3.32	50S ribosomal protein L30
AS87_05650	3.10	hypothetical protein
AS87_07815	3.56	50S ribosomal protein L18
AS87_07805	5.21	30S ribosomal protein S8
AS87_07850	3.05	30S ribosomal protein S13
AS87_02350	2.48	cystathionine beta-synthase
AS87_07760	2.79	50S ribosomal protein L22
AS87_04530	4.20	50S ribosomal protein L10
AS87_05170	2.79	30S ribosomal protein S7
AS87_03335	4.22	hypothetical protein
AS87_03730	0.06	acyl-protein systhetase, LuxE
AS87_04930	0.34	glycosyltransferase
AS87_05990	0.31	hypothetical protein

[a] Based on *R. anatipestifer* Yb2 genome (accession number: CP007204).

Supplementary Table S2. Primers used for real-time PCR analysis

Gene locus[a]	Forward primer sequences (5' - 3')	Reverse primer sequences (5' - 3')	Products (bp)
AS87_05650	GCGGGAGATGATGTGGTAGT	GAGTCAATGGCTCGCTGAGT	157
AS87_04530	AAAAGCAATGGAGCAAATGG	CAGGAGCATTAGCCGTCTCT	101
AS87_06160	ACGAAGCGTCGTTTTGAGAT	CAGCCTCCTCAATTCCAATC	130
AS87_03970	TTCCGCCTTCAGACTCCTTA	GCTTAGAGGGGAAGGTTTGG	210
AS87_07745	GGTTTTCCCAACTTGAAGTCC	TGTCTGATTTGAGAGGCGTTT	176
AS87_07845	CCTTGTCTTTGTTTAAATTTAGGG	GAGCATCAATTAAGAAGAGAAGTGC	103
AS87_06455	GCTTGTTGAGGCCAAGTTGT	TTTTCGCAGGAGCAGGTATT	130
AS87_06085	CTTTCGTTGGGCAATAGAGC	TAGCAAAACCCGTCCCATAG	181
AS87_07850	ACGTATTCCTCGTTGGCATC	TGAATTGGCTGCAATCAGAA	121
AS87_07760	GTGCAGGACGCAATCTTTTC	ATGATTGCCCTTCTTCTCCA	252
AS87_02350	CGACCCTCGCTCTTATTACG	GCAGAGCCAGAAAGTGTTCC	201
AS87_07855	GACCAGCTTCGTGAGCTACC	TCATATCCAAGCCACGTTCA	173
AS87_07860	TGCAGCAGCTCTAGTTTTAGCA	GCATGGGCCTAACAAAAGAA	232
AS87_07775	TCTTGCGATAGTCTTCCTCAAA	AGAAGTGAAAGCTACTTATGGGAAA	100
AS87_00900	AAGGCTGCAGAAAAAGCAAT	CTCCACAGCCAGGGAAATAA	106
AS87_07790	GGTTTGCAGCTGAAGGCTTA	GGAAGCAAAGGAGAAGTTCTTG	112
AS87_07830	GGCATTTGTCCTCCTTCAAA	GGACAAGGAAGTGGAAAAGG	104
AS87_07815	ACCCTGCAGCAATAGCTTTC	AAGTTTCCGGAACTCAAGCA	204
AS87_07805	TGCTGGTTTACCAGTTTGTTTG	GGAAATTCCTGCATCGAAAA	148
AS87_05175	CACACTTGCCAAGAAGAGCA	AAGCACCACGAACAATGTGA	266
AS87_04540	GGTGGTGCTGCTAATCCTTC	CACAGCTGCAGGAGGAGTTT	189
AS87_05170	CTTCCAAATTCCGATGCCTA	TCTTTTTGAAGGCAGCACCT	161
AS87_02865	CTTTTCAGCAGGACCACCAC	TATCTAGCGACTGCGGAAGG	102
AS87_04500	GGATAGGTTCGGCATCTTCA	TTGCTTGTTGACTCCCCTCT	129
AS87_07455	TATTCGTAGCGGCTGTTGCT	AGCACCAACACCTTCAGCAG	179
AS87_07825	AGCAGGTGTAGCTTCGTGCT	TGGCAACAATTAAAGTAAAACAAG	125
AS87_07870	TCATCACTTGCAGCAGAAGC	TTGCAGAGAGAAACGGTGGT	220
AS87_01795	CACCTCGGCTAAGGTAATGG	TGGGTTTTGGTTTTGAGGTC	165
AS87_05160	GGTGCTGTAGTGAATGGTCCT	TGAGCTGAAAGTTGGAACTGC	113
AS87_04930	ATTTGGGATGGGCTTAGGTT	CCCTTCTTTGTTCAGGCAAA	235
AS87_02265	GGGTAGATTTGGGTGTGTGG	GACGCATCTGTGCAGTAGGA	171
AS87_03335	ATCCGTTGACAAAACCGAAG	CCGTAGCATATTCGGAGGAA	155
AS87_05990	TGATGAGGATTGGATGTGGA	CCACCACCTTCCATAACTCC	153
AS87_03725	CGCCAAAAGCAACGATTTAT	CTTCTGGGTCGTTACGGAAA	155
AS87_03730	GAAGACCCGCTGTCGTATGT	CGCCCCAAAACTTGAAAATA	152
AS87_03735	AAGCTTTAGCGCTTCGAGTG	CAACACGGTACAACCACCAC	191

[a] Based on *R. anatipestifer* Yb2 genome (accession number: CP007204).
